# Relationship Between Trait Mindfulness and Sleep Quality in College Students: A Conditional Process Model

**DOI:** 10.3389/fpsyg.2020.576319

**Published:** 2020-09-29

**Authors:** Xiaoqian Ding, Xinshu Wang, Zirong Yang, Rongxiang Tang, Yi-Yuan Tang

**Affiliations:** ^1^College of Psychology, Liaoning Normal University, Dalian, China; ^2^Department of Gastroenterology, Affiliated Zhongshan Hospital of Dalian University, Dalian, China; ^3^Department of Psychological & Brain Sciences, Washington University in St. Louis, St. Louis, MO, United States; ^4^Department of Psychological Sciences, Texas Tech University, Lubbock, TX, United States

**Keywords:** mindfulness, sleep quality, negative emotions, neuroticism, conditional process model

## Abstract

Sleep quality can affect the physical and mental health, as well as the personal development of college students. Mindfulness practices are known to ameliorate sleep disorder and improve sleep quality. Trait mindfulness, an innate capacity often enhanced by mindfulness training, has been shown to relate to better sleep quality and different aspects of psychological well-being. However, how individual difference factors such as trait mindfulness relate to sleep quality remains largely unclear, which limits the optimization and further application of mindfulness-based intervention schemes targeting the improvement of sleep quality. In this study, we aimed to investigate how negative emotions and neuroticism may influence the relationship between trait mindfulness and sleep quality. A conditional process model was built to examine these relationships in 1,423 Chinese young adults. Specifically, the conditional process model was constructed with trait mindfulness as the independent variable, sleep quality as the dependent variable, negative emotions as the mediating variable, and neuroticism as the moderating variable. Our results showed that negative emotions mediated the link between mindfulness and sleep quality and that neuroticism had a moderating effect on the relationship between mindfulness and sleep quality. Together, these findings suggested a potential mechanism of how trait mindfulness influences sleep quality, provided a therapeutic target for which mindfulness-based interventions may act upon to improve sleep quality, and offered a basis for prediction of different intervention effects among individuals.

## Introduction

College students are important reserve talents for the construction of a country, and promoting their physical and mental health has been the goal of society. Sleep quality of college students is closely related to their health and personal development (Hsu et al., 2014; Lin et al., 2018; Li et al., 2019). A survey shows that from 18.7 to 21.4% of college students in China have sleep problems (Zhang et al., 2019), which manifest as falling asleep difficultly, low sleep efficiency, and severe effect on the study and life the next day. The high incidence of poor sleep and associated negative health consequences suggest the need for effective behavioral sleep interventions and a better understanding of the processes and mechanisms that affect the sleep quality of college students (Semplonius and Willoughby, 2018; Shallcross et al., 2019).

Mindfulness has been described as a non-reactive, non-judgmental, and present-centered awareness, in which each thought, feeling, or sensation is acknowledged and accepted as it is (Bishop et al., 2004). Some suggested that mindfulness is similar to character advantages in positive psychology and is often referred to as a trait that exists naturally and varies within the population, or as a state of consciousness that can be developed with mindfulness training (Davidson, 2010). However, mindfulness-based interventions have been effective in producing positive mental health outcomes, such as reducing physiological symptoms related to anxiety, levels of self-perceived stress, alleviating symptom severity of patients with mental illness (Choo et al., 2019), and improving inflammatory biomarker levels in older adults with mild cognitive impairment (Ng et al., 2020). These growing evidences indicate neurobiological effects and mechanism of mindfulness-based interventions. In recent years, growing lines of research show that higher trait mindfulness is related to better quality of sleep. For example, a higher level of trait mindfulness is significantly related to less sleep disturbance (Garland et al., 2013) and better sleep quality (Bogusch et al., 2016). One cross-sectional study showed that higher trait mindfulness of undergraduate students is associated with better self-reported sleep quality and reduced daytime sleepiness, presleep arousal, and dysfunctional beliefs about sleep (Howell et al., 2010). These findings suggest that trait mindfulness may be an important component in influencing individual’s sleep quality. Relatedly, Ong et al. (2012) proposed a specific metacognitive model of insomnia to explain the mechanisms by which mindfulness-based interventions positively impact subjective and objective sleep. The model proposed that the ability to accurately observe one’s internal and external experience may allow for more flexible responses to sleep difficulty by allowing the individual to disengage from their daily concerns and strivings. Shallcross et al. (2019) further offered an integrative model of sleep disturbance whereby key risk factors for compromised sleep quality and quantity are targeted through mindfulness practices.

While these theoretical considerations and burgeoning evidence studies have established the relationship between mindfulness and sleep quality, the precise mediating (i.e., how trait mindfulness relates to sleep quality) and moderating (i.e., how individual differences impact the relationships) mechanisms underlying the association between trait mindfulness and sleep quality remain largely unexplored.

### Negative Mood, Trait Mindfulness, and Sleep Quality

Many studies have shown that depression and anxiety can predict poor sleep quality (Demirci et al., 2015) and that higher-level negative emotions result in worse quality of sleep (Guo and Sun, 2016). Moreover, existing research shows that the main predisposing factors for insomnia are automatic activation of negative emotions or cognition before sleep (Harvey et al., 2002; Fernández-Mendoza et al., 2010). For example, according to the theory of embodied cognition (Mike, 2015) and cognitive behavioral therapy for insomnia (Blake et al., 2017), negative emotion before going to sleep may induce unreasonable rumination of thinking (Mike, 2015), thereby promoting excessive awakening of negative emotion and making it difficult to fall asleep (Blake et al., 2017). Thus, driven by negative emotions, a vicious circle of “individuals experience negative emotions as soon as they are ready to fall asleep, and insomnia as soon as they experience negative emotions” is initiated (Harvey et al., 2002; Fernández-Mendoza et al., 2010).

Many recent studies show that mindfulness-based interventions have a positive effect on emotion regulation (Gucht et al., 2019), including improvement in positive emotions and reduction in negative emotions (Erisman and Roemer, 2010; Ding and Tang, 2012; Ding et al., 2014a,b). For example, mindfulness meditation is effective for decreasing long-lasting maladaptive cognitive content and affective symptoms related to depression and anxiety (Ramel et al., 2004). Relatedly, trait mindfulness is negatively associated with negative affectivity, suggesting that trait mindfulness may be conducive to the awareness, acceptance, and elimination of negative emotions and negative thinking (Brown and Ryan, 2003). Together, these studies support the notion that mindfulness could facilitate emotion regulation, which may lead to reduced negative mood and increased positive affect.

Given that sleep problems are closely related to negative emotions, and mindfulness is associated with negative emotions, we speculate that trait mindfulness may exert its influence over sleep quality through negative emotions.

**Hypothesis 1**. Negative emotions mediate the relationship between mindfulness and sleep quality.

### Neuroticism, Trait Mindfulness, and Sleep Quality

Neuroticism, a major personality dimension (Gong, 1984; Matthews and Gilliland, 1999; Ding et al., 2015), is negatively associated with mindfulness in the general population (Giluk, 2009; Barnhofer et al., 2011; Hanley, 2016). It is possible that neurotic individuals inherently pay more attention to negatively personal feelings and cannot maintain awareness of their immediate experience (John and Srivastava, 1999), which may manifest as low level of mindfulness. Furthermore, this innate level of neuroticism may predispose individuals to negative affectivity, thereby influencing the effect of trait mindfulness on sleep quality. Our previous regression study showed that individuals with more emotional stability (meaning lower neuroticism) responded more favorably to the short-term mindfulness training in improving creative performance, whereas higher-neuroticism individuals failed to improve (Ding et al., 2015). In addition, according to the mindfulness intervention integrative model of sleep disturbance (Shallcross et al., 2019), adolescents with high level of neuroticism tend to pay more attention to negative stimuli (John and Srivastava, 1999), which may contribute to the maintenance of sleep disturbance by directing selective attention toward internal/external sleep-related threat cues, such as rumination, distorted perceptions, negative thoughts, and/or beliefs about sleeplessness. In contrast, for adolescents with low level of neuroticism, their attentional control should flexibly enable them to disengage more from negative perceived bias of sleep quality (William and Moroz, 2009; Harvey et al., 2014). Hence, individuals with high neuroticism may be inherently more susceptible to sleep problems (Trnka et al., 2012; Harvey et al., 2014; Kim et al., 2015; Yuan et al., 2018) and would have more difficulty in self-adjusting in order to fall asleep quickly. Therefore, another aim of the present study is to determine if the relationship between mindfulness and sleep quality would vary as a function of neuroticism level.

**Hypothesis 2.** Neuroticism moderates the relationship between mindfulness and sleep quality.

**FIGURE 1:**
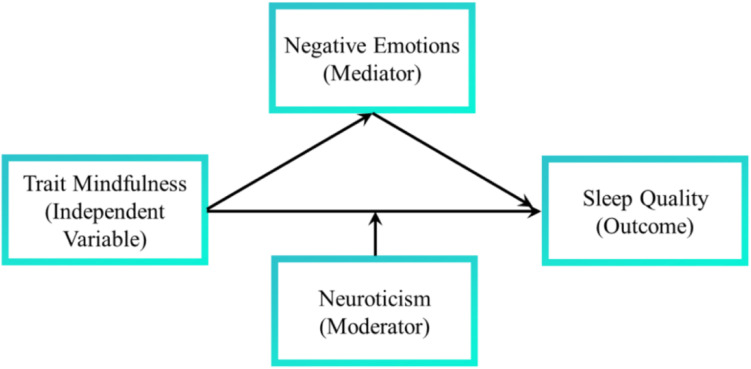
The proposed model.

Taken together, in a large sample, we aim to examine a conceptual model (Figure 1) in which, first, negative emotions mediate the relationship between trait mindfulness and sleep quality; second, the direct relationship between trait mindfulness and sleep quality is moderated by neuroticism.

## Materials and Methods

### Participants

Participants were selected by simple random sampling from several universities in Liaoning Province, China. A total of 1,528 participants were assessed using questionnaires, with 1,423 participants having usable data. Participants ranged from 17 to 23 years old [standard deviation (SD) = 0.84, mean = 19.57 years], and about 72% of the participants were females.

### Measures

#### Mindful Attention Awareness Scale

To assess individual differences in the frequency of attention to and awareness of present-moment experience, we used the Chinese version of Mindful Attention Awareness Scale (MAAS) (Deng et al., 2012) translated and revised from the scale of Brown and Ryan (2003). This scale consists of 15 items (e.g., “I find it difficult to stay focused on what is happening”). The participants rated each item on a 6-point scale ranging from 1 = *almost always* to 6 = *almost never*, with higher scores reflecting more mindfulness. For this study, the measure demonstrated high reliability (Cronbach α = 0.91).

#### Profile of Mood States

To assess transient and distinct mood states, we used the Chinese version of Profile of Mood States (POMS) (Wang et al., 2000) translated and revised from the scale of Mcnair et al. (1971). This 65-item scale evaluates six mood factors: Tension–Anxiety, Depression–Dejection, Anger–Hostility, Fatigue–Inertia, Vigor–Activity, and Confusion–Bewilderment. Participants rated each item on a 5-point scale ranging from 0 = *not at all* to 4 = *extremely*. The total mood disturbance (TMD) score is calculated by adding the scores for five mood factors (Tension–Anxiety, Depression–Dejection, Anger–Hostility, Fatigue–Inertia, and Confusion–Bewilderment) and subtracting the score for Vigor–Activity and then adding 100. The TMD score is a summary measure of negative emotion, with higher scores reflecting worse mood states (Wang et al., 2000). For this study, the measure showed high reliability (Cronbach α = 0.83).

#### Pittsburgh Sleep Quality Index

To assess sleep quality and discomfort for the past month, we used the Chinese version of Pittsburgh Sleep Quality Index (PSQI) (Liu et al., 1996) translated and revised from the scale of Daniel et al. (1989). PSQI consists of 19 self-rated questions and 5 questions rated by the bed partner or roommate. These self-rated items are grouped into seven component scores, each weighted equally on a 0- to 3-point scale. The seven component scores are then summed to yield a global PSQI score ranging from 0 to 21, and higher scores indicate worse sleep quality. For this study, the measure displayed high reliability (Cronbach α = 0.70).

#### Eysenck Personality Questionnaire

The emotional stability of a person was measured by the Chinese version of the N Scale of Eysenck Personality Questionnaire (EPQ) (Gong, 1984) translated and revised from the scale of Eysenck and Eysenck (1975). It applies to people older than 16 years. This N scale of EPQ consists of 24 items. The participants rated each item as “yes” or “no.” In this N scale, a high score indicates higher neuroticism, whereas a low score indicates higher stability. For this study, the measure demonstrated high reliability (Cronbach α = 0.90).

### Procedure

All materials and procedures were approved by the Ethics in Human Research Committee of the first author’s university. The simple random sampling was applied to select the target university. The data were collected online through the psychological test system for college students in October 2019. Importantly, the authenticity, independence, and integral nature of all answers were emphasized to the participants. Informed consent was obtained from the participants. All participants received a gift as compensation for their time.

### Statistical Analyses

Data entry, management, and descriptive statistics were performed using SPSS 25.0. First, descriptive analysis of the main variables and Pearson correlation analysis were performed (Wang et al., 2017). Second, the independent variable, the intermediate variable, the adjusted variable, and the dependent variable in the corresponding boxes were selected in turn using the plug-in PROCESS, and Model 5 was selected with the sample size set to 5,000 (Wang et al., 2017; Hayes and Rockwood, 2020). Next, the non-parametric percentile bootstrap method for bias correction was selected, and 95% confidence interval was calculated. The grouping conditions were set to mean and mean ± 1 SD (Wang et al., 2017; Hayes and Rockwood, 2020).

## Results

### Preliminary Analysis

Correlation analysis was performed on gender, age, trait mindfulness, negative emotions, sleep quality, and neuroticism. Means, SDs, and zero-order correlations for all study variables are presented in Table 1. As expected, college students with higher mindfulness or with lower negative emotions had better sleep quality. Additionally, college students with higher trait mindfulness had fewer negative emotions. Further, college students with higher neuroticism had lower mindfulness, higher negative emotions, and worse sleep quality.

**TABLE 1:** Descriptive statistics and related analysis results of each variable.

Variables	Mean	SD	1	2	3	4	5	6
1. Gender	0.72	0.45	1					
2. Age	19.57	0.84	−0.11**	1				
3. Mindfulness (MAAS)	62.39	13.53	0.01	0.00	1			
4. Negative mood (POMS)	140.39	33.60	−0.09**	0.09**	−0.43**	1		
5. Sleep quality (PSQI)	4.65	3.12	0.03	0.04	−0.24**	0.38**	1	
6. Neuroticism (EPQ-N)	12.53	6.37	0.06*	–0.04	−0.42**	0.51**	0.29**	1

N = 1,423. Gender as a dummy variable, male = 0, female = 1, and the average value of gender indicates the proportion of females. SD, standard deviation; **p < 0.01.

### Influence of Trait Mindfulness on Sleep Quality of College Students: A Conditional Process Model

The bootstrap mediation effect test method was adopted, and the conditional process model (Model 5) proposed by Andrew F. Hayes was used in tests (Hayes and Rockwood, 2020):

M=i+1aX1+eMY=i+2c′X1+c′W2+c′X3W+bM+eY

where *X*, *Y*, *W*, and *M* represent trait mindfulness, sleep quality, neuroticism, and negative emotions, respectively. Results yield the following equations:

$$\begin{array}{c} M\,\wedge =-0.43\,X\\ Y\,\wedge =0.02-0.08\,X+0.11\,W+0.06\,X\,W+0.28\,M \end{array}$$

The combination of the two and the overall prediction equation is *Y*∧ = 0.02 - 0.20*X* + 0.11*W* + 0.06 *XW*. The relationships between mindfulness and negative emotions, mindfulness and sleep quality, and negative emotions and sleep quality were all significant, so partial mediation relationships exist among mindfulness, negative emotions, and sleep quality (Table 2). Furthermore, with sleep quality as the dependent variable, the interaction between mindfulness and neuroticism was significant, indicating that neuroticism has a moderating effect on the relationship between mindfulness and sleep quality.

**TABLE 2:** Conditional process model effect.

Variables	Results							
	*M*				*Y*			
		Coefficient	SE	*P*		Coefficient	SE	*P*
*X*	a_1_	−0.43	0.02	0.00	c’_1_	−0.08	0.03	0.01
*M*		—	—	—	b	0.28	0.03	0.00
*W*	a_2_	—	—	—	c’_2_	0.11	0.03	0.00
*X* × *W*	a_3_	—	—	—	c’_3_	0.06	0.02	0.00
Constant	i_1_	0.00	0.02	1.00	i_2_	0.02	0.03	0.35
	*R*^2^ = 0.18				*R*^2^ = 0.16			
	*F* = 318.13, *P* = 0.00				*F* = 69.91, *P* = 0.00			

According to *M* + SD, *M*, and *M* - SD, neuroticism was divided into three levels of low, medium, and high, and the direct influence of mindfulness on sleep quality was analyzed. The direct impact of mindfulness on sleep quality was significant in both the low-and medium-neurotic groups and was stronger in the low-neuroticism group (Table 3). In high-level neurotic populations, the direct impact of mindfulness on sleep quality was insignificant. The confidence intervals of the bootstrap test were (−0.22, −0.07), (−0.13, −0.03), and (−0.08, 0.05). The first two did not contain 0, and the latter contained 0.

**TABLE 3:** Effects of mindfulness on sleep quality at different levels of neuroticism.

*W*	Direct effects on sleep quality		
	Effect	*SE*	*P*
−1.00	−0.14	0.04	0.00
0.00	−0.08	0.03	0.00
1.00	−0.02	0.03	0.58

**FIGURE 2:**
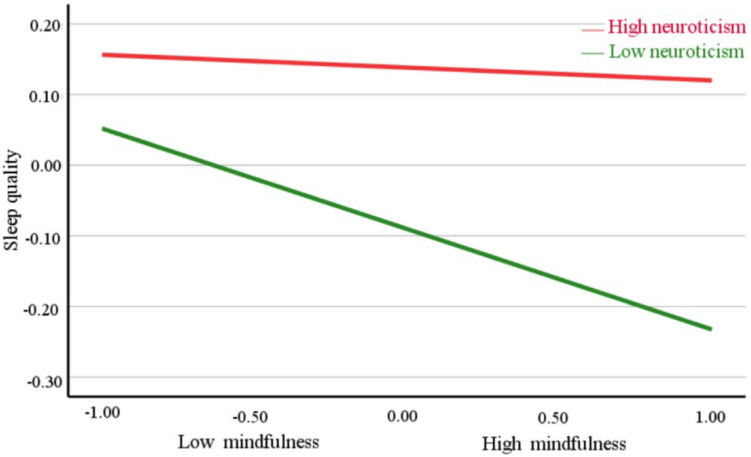
Effects of mindfulness (MAAS score) and neuroticism (EPQ-N score) on sleep quality (PSQI score). Functions are graphed for two levels of neuroticism: the red and green lines represent the standard deviations above and below the mean, respectively. Sleep quality of low-neurotic individuals increases as the level of mindfulness increases, but sleep quality of high-neurotic individuals barely changed.

Mindfulness and neuroticism were divided into low and high groups according to M - SD and M + SD, and the impact of mindfulness on sleep quality at different levels of neuroticism was investigated via analysis of variance. Also, simple effect analysis charts were drawn. As the level of mindfulness increased, sleep quality in the high-level neuroticism group remained unchanged, whereas sleep quality in the low-level neuroticism group decreased (Figure 2).

## Discussion

The relationship between trait mindfulness and sleep quality has been reliably shown by previous research (Howell et al., 2010; Garland et al., 2013; Bogusch et al., 2016). However, questions concerning the underlying mediating and moderating mechanisms remained largely unclear. The current study tested whether trait mindfulness would be indirectly associated with sleep quality through negative emotions and whether this relationship between trait mindfulness and sleep quality would be moderated by neuroticism in college students. Our results indicated that the impact of trait mindfulness on sleep quality can be partially explained by negative emotions. That is, trait mindfulness negatively predicted negative emotions, and in turn, negative emotions predicted worse sleep quality. Furthermore, the relationship between trait mindfulness and sleep quality was moderated by neuroticism, such that the path from trait mindfulness to sleep quality was stronger in individuals with lower neuroticism, but the relationship becomes less pronounced in individuals with higher neuroticism. In other words, it is possible that trait mindfulness may be a protective factor against sleep problems for college students with low neuroticism, whereas in college students with high level of neuroticism, the predisposition toward negative affectivity may attenuate such protective influence by interfering with the mindful non-judgmental and non-reactive awareness critical for improving sleep quality.

### The Mediating Role of Negative Emotions

We found that the impact of trait mindfulness on sleep quality can be partially explained by negative emotions, such that college students with higher mindfulness have fewer negative emotions, which then leads to better sleep quality. This result suggests that reduced negative emotions can be one of the explanatory mechanisms for why college students with high level of trait mindfulness are less susceptible to sleep disorders. Further, these findings support the metacognitive model of insomnia (Ong et al., 2012), which proposes that the ability to accurately observe one’s internal and external experience (higher trait mindfulness) may allow for more flexible responses to sleep difficulty by allowing the individual to improve the emotion regulation. In past research of sleep disturbances, clinical psychologists paid attention to the effects of mindfulness-based intervention, whereas psychiatrists emphasized pharmaceutical approaches to improve emotion. These two research fields have independently developed without examining the potential association between mindfulness and negative emotions and their potential implication for sleep quality. Our results provide support for an integrated model, in which trait mindfulness serves as a protective factor against negative emotions, leading to decreases in rumination or mood-related disturbances that ultimately translate into improvement in sleep quality.

In addition to the mediation effect, each of the separate associations in the mediation model is noteworthy. For the first stage of the mediation process (trait mindfulness → negative emotions), our finding supports the notion that mindfulness could facilitate successful emotion regulation and that a higher level of mindfulness is related to lower negative mood. This finding is consistent with previous mindfulness-based interventions studies (Erisman and Roemer, 2010; Ding and Tang, 2012; Ding et al., 2014a,b), indicating that the increasing frequency of attention to and awareness of present-moment experience can play a vital role in improving emotion regulation (Ramel et al., 2004; Erisman and Roemer, 2010; Gucht et al., 2019). For college students with high level of mindfulness, they also tend to have lower negative affect, which in turn could protect them from sleep problems. For the second stage of the mediation model (negative emotions → sleep quality), our results indicated that negative emotions were negatively associated with good sleep quality. This finding is also in line with the theory of embodied cognition (Mike, 2015) and cognitive behavioral therapy for insomnia (Blake et al., 2017), which propose that people with maladaptive emotional response are more likely to initiate a vicious circle between negative emotions and sleeplessness (Harvey et al., 2002; Fernández-Mendoza et al., 2010). In addition, this finding is consistent with previous studies showing that negative emotion is a predisposing factor for college students’ insomnia (Demirci et al., 2015; Mike, 2015; Guo and Sun, 2016).

### The Moderating Role of Neuroticism

The second goal of this study was to examine whether neuroticism would moderate the direct link between trait mindfulness and sleep quality. The results revealed that neuroticism moderated the path between trait mindfulness and sleep quality. The relationship between trait mindfulness and sleep quality was significant for students with low level of neuroticism, however, it was not significant for students with high level of neuroticism.

One explanation might be that high neuroticism influences cognitive–affective processes of mindfulness and weakens the effect of trait mindfulness on sleep quality. Suls and Martin (2005) proposed that neurotic cascade is formed by several interconnected processes, such as hyperreactivity to negative stimuli, appraisal of stressful events as more threatening, and rumination. Each maladaptive coping strategy reinforces each other and creates difficulties for maintaining awareness of immediate experience (John and Srivastava, 1999), which may attenuate or even offset the positive impact of trait mindfulness on sleep quality. Another explanation might be that, compared with low-neurotic individuals, individuals with high neuroticism tend to pay more attention to negative stimuli, further exacerbating the cognitive and behavioral vulnerabilities associated with poor sleep and preventing attentional resources from being allocated to mindful awareness and emotion regulation processes (William and Moroz, 2009; Harvey et al., 2014; Shallcross et al., 2019).

Moreover, the moderating effect of neuroticism may explain some inconsistent effects of mindfulness-based intervention on sleep quality (Ding et al., 2015; Kanen et al., 2015). Based on a meta-analysis of 575 individuals across 16 studies, it was found that 82.09% of the mindfulness-based interventions were associated with sleep improvement, suggesting a possibility that individual differences in neuroticism may play a role in these differential effects. Relatedly, our previous individual difference study of creative improvement following mindfulness meditation showed that the individuals with lower neuroticism responded more favorably to the short-term mindfulness training in improving creative performance, whereas higher-neuroticism individuals failed to improve (Ding et al., 2015). These results also suggest that neuroticism can be regarded as a promising indicator of who would likely benefit more from mindfulness-based interventions.

### Implications

First, this study shows that people with higher trait mindfulness sleep better. As reported, individuals with higher trait mindfulness pay attention to the physical cues of sleepiness and choose to act on these cues in a way consistent with healthier sleep patterns (Andrew et al., 2008). Previous studies have found that attention to self-physiology promotes sleep. Moreover, the non-alert attention of mindfulness affects mood and attention network efficiency, and adjustment of brain activity is significantly better than the alerting attention of relaxation training (Tang et al., 2019). The mindful attention state may improve sleep through the regulation of emotions.

Second, this study suggests an internal mediating mechanism of negative emotions on the relationship between sleep quality and mindfulness, providing a potential intervention target or mechanism of mindfulness-based interventions that seek to promote sleep quality. For instance, future mindfulness-based interventions could improve sleep quality by focusing on reducing negative emotions through increasing trait mindfulness. Sleep health education and emotion management are important parts of mental health education in colleges and universities. Based on the results of this study, the mindfulness training may serve as a sleep enhancement method and be part of mental health courses in college students. Teachers could also help college students reduce negative emotions through mindfulness training to improve their sleep quality. Our results may further enrich the ways and contents of mental health education for college students and provide reference for the improvement and development of sleep health education.

Third, this study demonstrates that people with lower neuroticism have higher levels of mindfulness. Importantly, as a trait-like psychological construct, trait mindfulness can be enhanced through meditation or mindfulness training (Tang et al., 2016), which implies that improving trait mindfulness might be an effective intervention strategy for sleep quality in low-neuroticism individuals. Neuroticism may be useful tools to predict individual variations in the improvement of sleep quality following mindfulness training.

With the technological advances, in recent years, the traditional mindfulness programs have been turned more and more into digital applications, which promise increased spatial and temporal flexibility, as well as an individual use. Therefore, there is an urgent need to take into consideration designing a conceptualized application of mindfulness interventions to improve sleep quality. A 5 min mindfulness application to relieve distress and suffering among palliative patients was conceptualized and tested in pilot and randomized trials and provided a good example to control for placebo effects (Zhang et al., 2017). It is time to design mindfulness interventions and relieve insomnia suffering among college students with poor sleep quality or sleep disorder patients. For example, the applications may be designed to measure the emotional stability of the users to predict individual variations in the improvement of sleep quality following mindfulness interventions.

### Limitations and Future Directions

Some limitations should also be considered in interpreting the results of this study. First, this is a cross-sectional study. The data of the main research variables were collected at the same time. Although the analysis verified the research hypotheses, it cannot prove the cause-and-effect relationship. Future longitudinal studies, behavior experiments, and other methods can be designed to further explore the internal mechanism underlying the effects of mindfulness on sleep quality. Second, only college students were selected, which may not be universally representative of the samples. Future research can expand the scope of sample selection. Third, neurophysiological evidence could potentially be considered as objective evidence for examining the effect of mindfulness (Choo et al., 2019). Brain activity and connectivity may also influence sleep quality, which should also be considered in future mediation and moderation models.

## Conclusion

College students with higher mindfulness traits have better sleep quality. Negative emotions can mediate the relationship between mindfulness and sleep quality. Furthermore, neuroticism moderates the relationship between mindfulness and sleep quality. In particular, low-level neurotic college students showed stronger positive relationship between mindfulness and sleep quality, suggesting that improving trait mindfulness through proper mindfulness training may help to reduce negative emotions and improve sleep quality.

## Data Availability Statement

The raw data supporting the conclusions of this article will be made available by the authors, without undue reservation, to any qualified researcher.

## Ethics Statement

The studies involving human participants were reviewed and approved by the Liaoning Normal University ethics committee. The patients/participants provided their written informed consent to participate in this study.

## Author Contributions

XD and XW designed and conducted the study. XD, XW, and RT analyzed the data. XD, XW, RT, ZY, and Y-YT wrote the manuscript. All authors contributed to the article and approved the submitted version.

## Conflict of Interest

The authors declare that the research was conducted in the absence of any commercial or financial relationships that could be construed as a potential conflict of interest.
